# Rotational radiofrequency‐based technology leads to adipose tissue reduction and contouring effect in the thighs, abdomen, and flanks

**DOI:** 10.1111/jocd.16431

**Published:** 2024-07-01

**Authors:** Alejandra Fernández Santos, Alejandra Iglesias Fernández, Lorena Suárez Fernández, Leticia Huergo Zapico, Susana Valero Freitag

**Affiliations:** ^1^ Termosalud SL Gijón Spain

**Keywords:** dermal thickness, radiofrequency, skin structure, subcutaneous fat

## Abstract

**Background:**

Excess body adiposity and skin irregularities represent a major problem in today's society. Currently, radiofrequency‐based devices constitute an increasingly popular medical‐aesthetic application and a powerful non‐invasive method to combat this problem.

**Objective:**

This study aims to determine the efficacy and safety of the high‐frequency device Zionic™ (Termosalud, Inc., Gijón, Spain) in reducing body contouring and improving skin appearance in the abdomen/flanks or thighs.

**Materials and Methods:**

Thirty nine individuals were treated with the Zionic™ device in the abdomen/flanks or thighs. A total of 8 sessions of 40–50 min each, 72/96 h apart were performed. At baseline, body measurements were taken including photographs, body contours, and ultrasound scans to assess dermal thickness, dermal echogenicity, and subcutaneous fat thickness. The measurements were repeated after the eight sessions for comparison and analysis. Quantitative data was complemented with a customized survey to evaluate participants satisfaction level.

**Results:**

At the follow‐up visit, a significant average reduction of 3% in abdomen/flanks circumferences and 2% in thighs contours was noticed. Abdomen/flanks and thighs subcutaneous fat layer thickness was significantly reduced by 8% and 6%, respectively. Dermal echogenicity changes, related to skin collagen content and organization, showed non‐significant increasing tendencies of 7% for abdomen/flanks and 8% for thighs. Thighs dermal thickness was significantly increased by 6%. Results were associated to a high satisfaction level (80%) and no severe adverse events.

**Conclusion:**

Zionic™ treatment is a safe, effective, and well‐tolerated noninvasive procedure for body contouring and improvement of skin properties in abdomen, flanks, and thighs.

## 1 INTRODUCTION AND OBJECTIVE

Adipose tissue accumulation at specific anatomical locations, often referred to as localized adiposity, is a widespread aesthetic concern that can significantly impact an individual's self‐perception and well‐being.^1^, ^2^ While conventional methods such as diet and exercise can be effective in addressing general weight management, they may not effectively target localized fat deposits. In these instances, invasive procedures like liposuction have been considered a viable solution; however, their high cost, surgical risks, and extended downtime have led to a growing preference for noninvasive alternatives. Radiofrequency (RF)‐based devices have emerged as a leading noninvasive approach for body contouring, offering effective, safe, and minimally invasive treatments.^3^ This study aims to evaluate the safety and efficacy of the new generation monopolar RF device Zionic™ in reducing localized fat accumulation in the abdomen/flanks and thighs.

RF‐based devices, also known as diathermia or TECAR devices, work by delivering controlled heat to the deep layers of the skin, reaching temperatures between 41°C and 43°C when measured on the epidermis. This targeted heating stimulates dermal fibroblasts, promoting the synthesis of new extracellular matrix components, such as collagen, elastin, and hyaluronic acid (HA),^4^, ^5^ leading to improved skin elasticity and firmness. At the hypodermal level, thermal stimulation increases the metabolic rate of adipocytes, leading to the reduction of fat tissue by lipolytic and/or apoptotic means.^6^ High temperatures during RF treatments also enhance blood circulation and lymphatic drainage.^5^ This improved circulation helps to remove excess fluids and toxins from the treated area, further contributing to skin tightening and a reduction of skin irregularities such as those caused by cellulite. Additionally, increased blood flow delivers more oxygen and nutrients to the skin, promoting healing and regeneration.^2^, ^7^

The combined effects of collagen production induction, fat reduction, improved circulation, and lymphatic drainage make RF a comprehensive technology for body contouring. The treatments are well‐tolerated by most patients and can be tailored to individual needs and clinical goals.

Zionic™ (Termosalud, Inc., Gijón, Spain) is a new generation device combining monopolar radiofrequency (RF) energy with deep active rotational massage for noninvasive aesthetic treatments. Numerous studies support the efficacy of monopolar RF biostimulation in reducing fat tissue, improving skin appearance, and decreasing body circumferences.^8^, ^9^ In parallel, research has demonstrated that manual therapy and mechanical tissue massage promote circulation, eliminate waste products, oxygenate targeted tissues, and enhance skin texture.^10^ Therefore, Zionic™, which concurrently applies both RF and mechanical stimulation, represents a promising tool to address these aesthetic concerns.

This study meticulously assessed the safety and efficacy of Zionic™ in addressing localized fat accumulation in the abdomen/flanks and thighs. It aimed to shed light on the device's potential as a transformative solution for body contouring, offering a noninvasive, effective, and safe alternative to traditional methods.

## 2 MATERIALS AND METHODS

### 2.1 The device

Zionic™ is a rotational RF‐based device that applies a high‐frequency, low‐impedance alternating current to a circuit consisting of a rotational electrode and a return plate placed on one side of the treated area. The device allows adjustment of the direction, the speed of rotation, and the applied power (up to 200 W). An embedded infrared sensor monitors the temperature of the skin surface and guides the operator during treatment.

### 2.2 Subjects

In the study, 39 healthy volunteers, women, aged 23–68 years old, and Fitzpatrick skin Type II–III were considered. The eligibility of all participants was assessed according to the inclusion/exclusion criteria: candidates under the age of 18, over the age of 70 and/or presenting any of the treatment contraindications (Data S1) were not considered. Inclusion criteria comprised subjects between 18 years and 70 years old exhibiting accumulation and/or hypertrophy of adipose tissue in the area to be treated. The flow diagram of the participants throughout the trial was included in the supplementary material S2. Written informed consent was obtained from all included subjects after a complete explanation of the study procedure. The investigation was approved by the Research Ethics Committee of the Principality of Asturias.

### 2.3 Experimental procedure

A total of 8 sessions of 45–50 min treatment were applied on the thighs or abdomen/flanks separated by 72/96 h. During each of these sessions, a low impedance and high frequency RF current was applied in combination with the rotational massage. The treatment thermal aim was to achieve 41–43°C (hyperthermia) in the area and maintain it for 10 min. Therefore, movement was constant to cover all the area.

At baseline and at the final follow‐up visit (1 week after the last session), data collection (Table S3) was carried out by using the methods described below.

### 2.4 Circumference measurements

Body contour evaluation was performed by using a standardized measure tape. The subjects were in a standing standardized position and the measure tape was kept in constant contact with the subjects' skin. Contour measurements at three different heights of the abdomen/flanks (upper contour, middle contour and lower contour) and at two different heights of the thighs (upper contour and lower contour), were taken at baseline and at the follow‐up visit (Figure S4).^1^, ^11^

### 2.5 Photography

Standardized photographs were taken of participants before and after the study. A professional camera (Canon EOS 60D) was used under consistent lighting conditions. To ensure a standardized posture, participants stood on a fixed footprint template with their arms bent at chest height. Four photos were taken of each participant: front, left side, right side, and back. Before and after photos were combined using Photoshop software (24.1.1 version). No other image editing software was used.

### 2.6 Ultrasound measurements

At baseline and at the final follow‐up visit, ultrasound analyses of the dermal and hypodermal structure were performed using DermaLab® Combo probes (Cortex Technology, Denmark). For each subject, subcutaneous thickness, dermal thickness, and dermal echogenicity (DE) (as an indirect estimation of dermal collagen level) were assessed at three different points (Figure S4). Using the 10 MHz focused ultrasound probe, hypodermal subcutaneous thickness was measured as the distance between the deep dermis line and the deep fascia membrane. Dermal thickness and DE were assessed using a 20 MHz focused ultrasound probe. DE serves as a surrogate marker for collagen content. Collagen, a highly echogenic protein within the extracellular matrix, reflects ultrasound waves to a greater extent compared to surrounding structures. By quantifying echogenicity changes, researchers can indirectly assess dermal collagen levels. This noninvasive technique provides valuable insights into the structural integrity and physiological state of the dermis.^12^

### 2.7 Subject satisfaction assessment and adverse events

Satisfaction assessment was carried out 1 month after the final measurements. It consisted of a questionnaire prepared by Termosalud SL. Questions regarding participant demography, treatment performance, adverse effects occurrence, and satisfaction level (0–4: not satisfied; 5–6: satisfied; 7–10: very satisfied) were recorded according to a numerical scale or by selecting one of the pre‐established answers (Data S5).

### 2.8 Statistical analysis

All statistical analyses were performed with R software and Rcmdr package (R version 4.3.02023 and Rcmdr version 2.8–0). Descriptive analysis, including means, medians, ranges, and standard deviations were made to describe the improvement of the different parameters and measurements (body contours, fat layer thickness, dermal thickness, and DE). Normal distribution assumption was assessed with Kolmogorov–Smirnov test, while comparisons between before and after treatment (follow‐up) were performed using Paired sample *t*‐test or Wilcoxon's signed‐rank test for matched pairs. *p* <0.05 was considered statistically significant. The effect size to establish the differences between the two groups means was estimated for each significant statistical analysis. Cohen's *d* (*d*) formula was used for Paired sample *t*‐tests and R correlation coefficient (*r*) for Wilcoxon's signed‐rank tests. Cohen's *d* effect size was considered small (0.2 ≥ *d* ≤ 0.5; slight differences), moderate (0.5 ≥ *d* ≤ 0.8; medium differences) or large (*d* ≥ 0.8; high differences). R correlation coefficient was referenced as small (0.1 ≥ *r* ≤ 0.3; slight differences), moderate (0.3 ≥ *r* ≤ 0.5; medium differences) or large (*r* ≥ 0.5; high differences).^13^

## 3 RESULTS

### 3.1 Participant baseline characteristics

All the 39 participants involved in the clinical trial completed their treatments (Table 1): 64.10% in the abdomen/flanks (*n* = 25) and 35.90% in the thighs (*n* = 14). The age range included subjects between 23 and 68 years old (mean age of 47 years old). The average body weight reduction of the 39 participants, observed in the follow‐up visit, was 0.48 ± 1.13 kg.

**TABLE 1:** Demographic and clinical information of the subjects included in the clinical trial.

Parameter/measurement	Values/change (range)
Age range (years)	23–68
Gender (*n*)	
Female	39
Treatment area (*n*)	
Abdomen/flanks	25
Thighs	14
Body weight loss (Kg)	0.48 ± 1.13
Body contour reduction (cm)	
Abdomen/flanks contours	
Upper	2.55 (0.5–4.5)
Middle	2.50 (0.5–11.5)
Lower	2.75 (0.3–12)
Thighs contours	
Upper	1 (0.5–3.5)
Lower	2.12 (0.5–6)
Fat layer thickness reduction (%)	
Abdomen/flanks	6
Thighs	8
Skin echogenicity increase (%)	
Abdomen/flanks	7*
Thighs	8*
Skin thickness increase (%)	
Abdomen/flanks	–
Thighs	6

* *p* > 0.05.

### 3.2 Evaluation of abdomen/flanks and thighs circumference changes

Abdominal measurements showed a significant reduction at all the measuring points in the follow‐up session (Figure 1): 2.55 ± 1.36 cm (range: 0.5–4.5 cm) in the upper contour (*t* = 7.29, *p* < 0.01, *d* = 1.88‐high effect size‐); 2.50 ± 2.53 cm (range: 0.5–11.5 cm) in the middle contour (*W* = 190, *p* < 0.01, *r* = 0.88‐high effect size‐) and 2.75 ± 2.63 cm (range: 0.30–12 cm) in the lower contour (*W* = 300, *p* < 0.01, *r* = 0.88‐high effect size‐). The circumference reductions mentioned represented a decrease in body contours of 3%.

**FIGURE 1:**
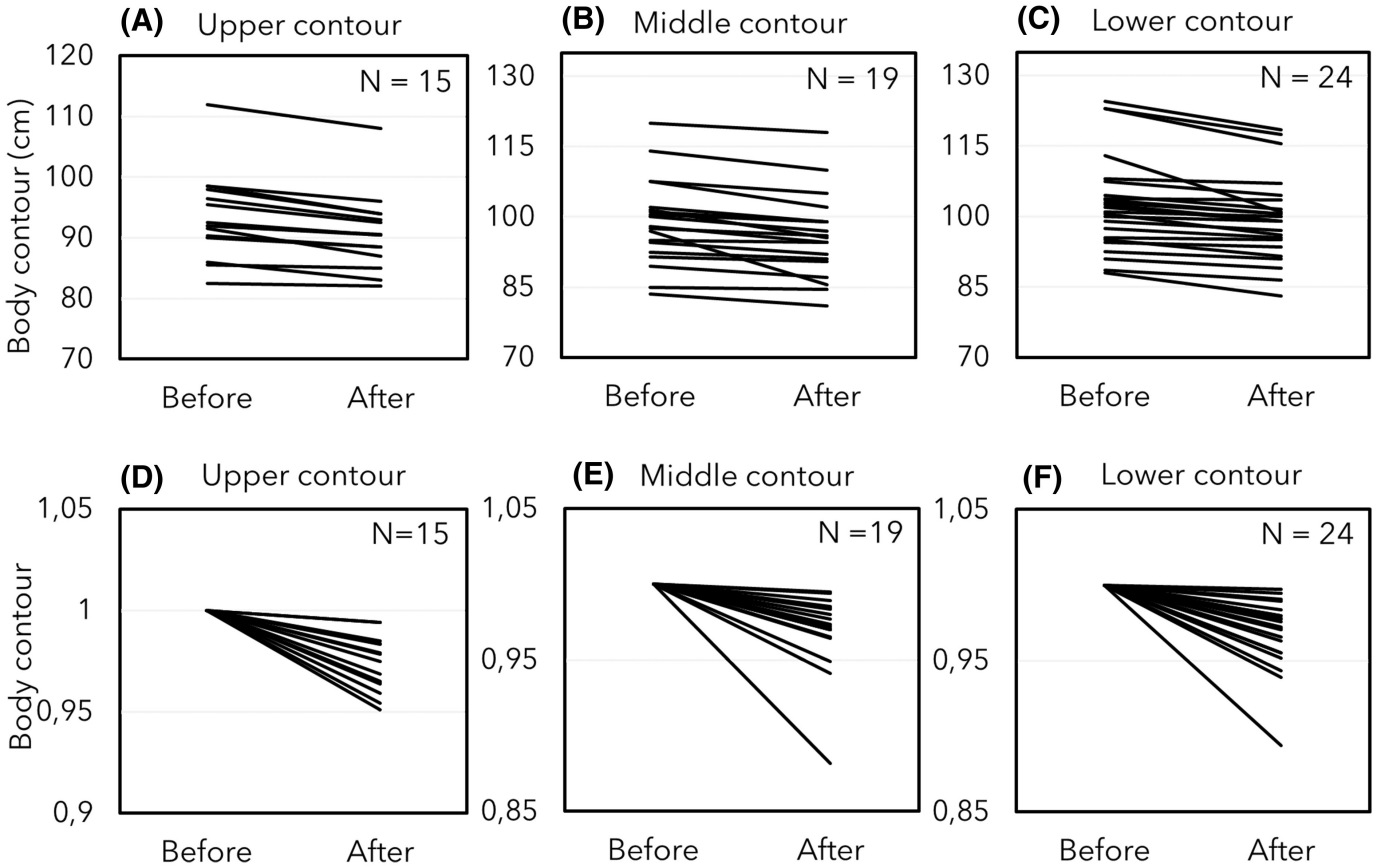
Abdomen/flanks' circumference assessment after treatment. Three reference heights were considered. Before and after body contour measurements (cm) at the (A) upper, (B) middle, and (C) lower points. Normalized representations of the contour changes in abdomen/flanks: (D) upper, (E) middle, and (F) lower heights.

Thigh circumferences (both, upper and lower contours) showed a significant reduction after the treatment (Figure 2). The upper circumference was reduced a mean value of 1 ± 0.99 cm (range: 0.5–3.5 cm), which represented a 2% decrease of circumference (*W* = 66, *p* < 0.01, *r* = 0.89‐high effect size‐). Lower measure showed a 2.12 ± 1.53 cm (0.5–6 cm; 4%) reduction comparing to baseline (*t* = 4.79, *p* < 0.01, *d* = 1.39‐high effect size‐).

**FIGURE 2:**
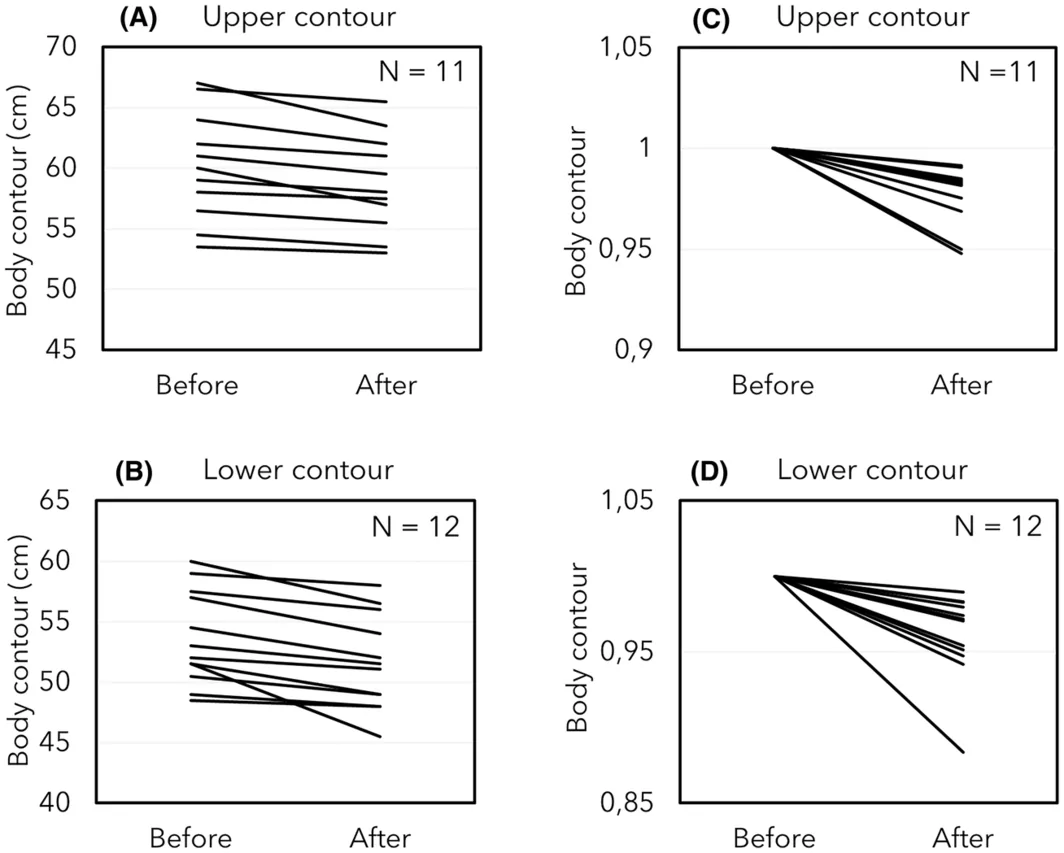
Thighs' circumference assessment after treatment. Two reference heights were considered. Before and after body contour measurements (cm) at the (A) upper and (B) lower points. Normalized representations of the contour changes in thighs: (C) upper and (D) lower heights.

### 3.3 Evaluation of subcutaneous fat layer thickness

Analysis of subcutaneous fat layer thickness (mm) in the abdominal area (Figure 3A) showed a significant mean decrease of 6% (1.32 ± 2.56 mm) (*W* = 2191, *p* < 0.01, *r* = 0.47‐moderate effect size‐), with a maximum reduction of 32%. In the thighs (Figure 3B) the mean reduction observed was 8% (1.06 ± 2.50 mm) (*W* = 723, *p* < 0.01, *r* = 0.65‐high effect size‐), reaching values of up to 33% in some very responsive individuals.

**FIGURE 3:**
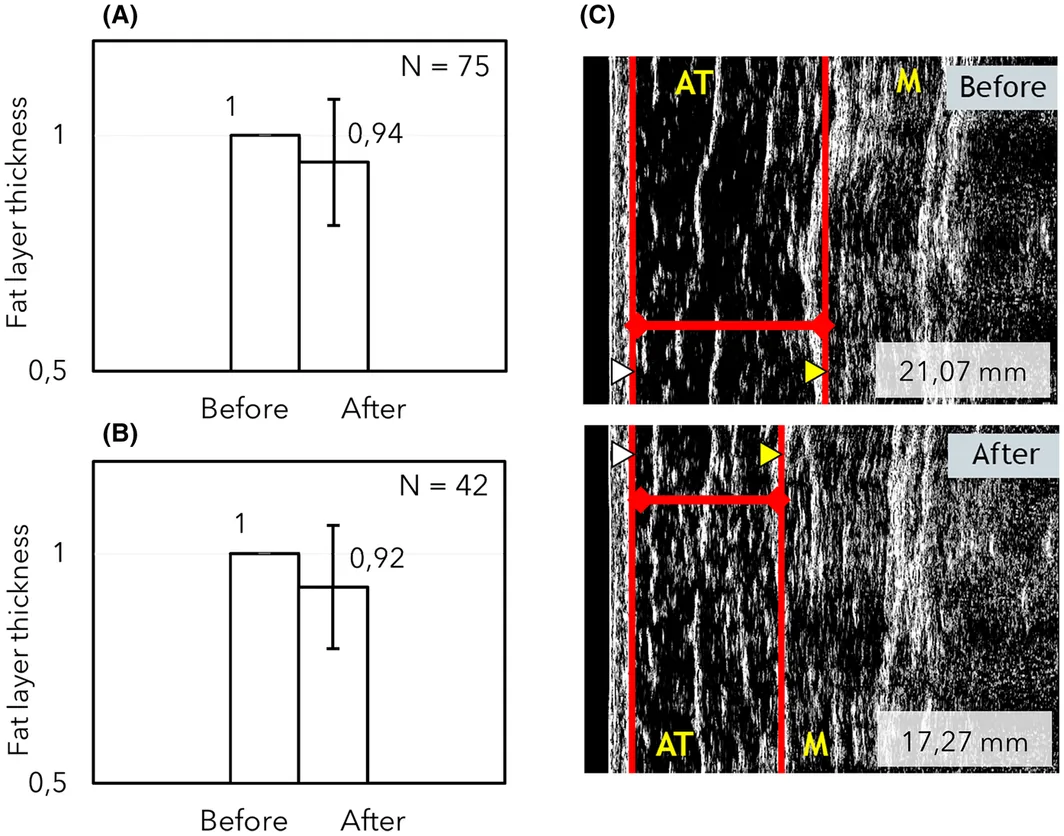
Before and after ultrasound measurements of subcutaneous fat layer thickness. Normalized representations of the fat thickness reduction in (A) abdominal and (B) thighs areas. (C) Example of subcutaneous ultrasound images; a reduction from 21.07 to 17.27 mm was observed after 8 Zionic™ sessions. ▷ Deep dermis line; AT: adipose tissue (hypodermis); Deep fascia membrane. M: muscle.

### 3.4 Evaluation of dermal structure and thickness

DE changes between baseline and at the follow‐up visit were assessed. This value was used as an indirect measure of the structural collagen present in the dermis. In both abdominal and thighs areas, DE increased in the follow‐up measurements. In abdomen/flanks an average improvement of 7% (*W* = 129, *p* = 0.38) was noted (Figure 4A). In the thighs, DE increase represented an 8% (*t* = −1.69, *p* = 0.11) (Figure 4B). Consistent with these results, thighs dermal thickness showed a significant mean increase of 6% (72.23 ± 124.44 μm) from baseline values (*t* = − 2.17, *p* < 0.05, *d* = −1‐high effect size). In the abdominal area, dermal thickness did not change.

**FIGURE 4:**
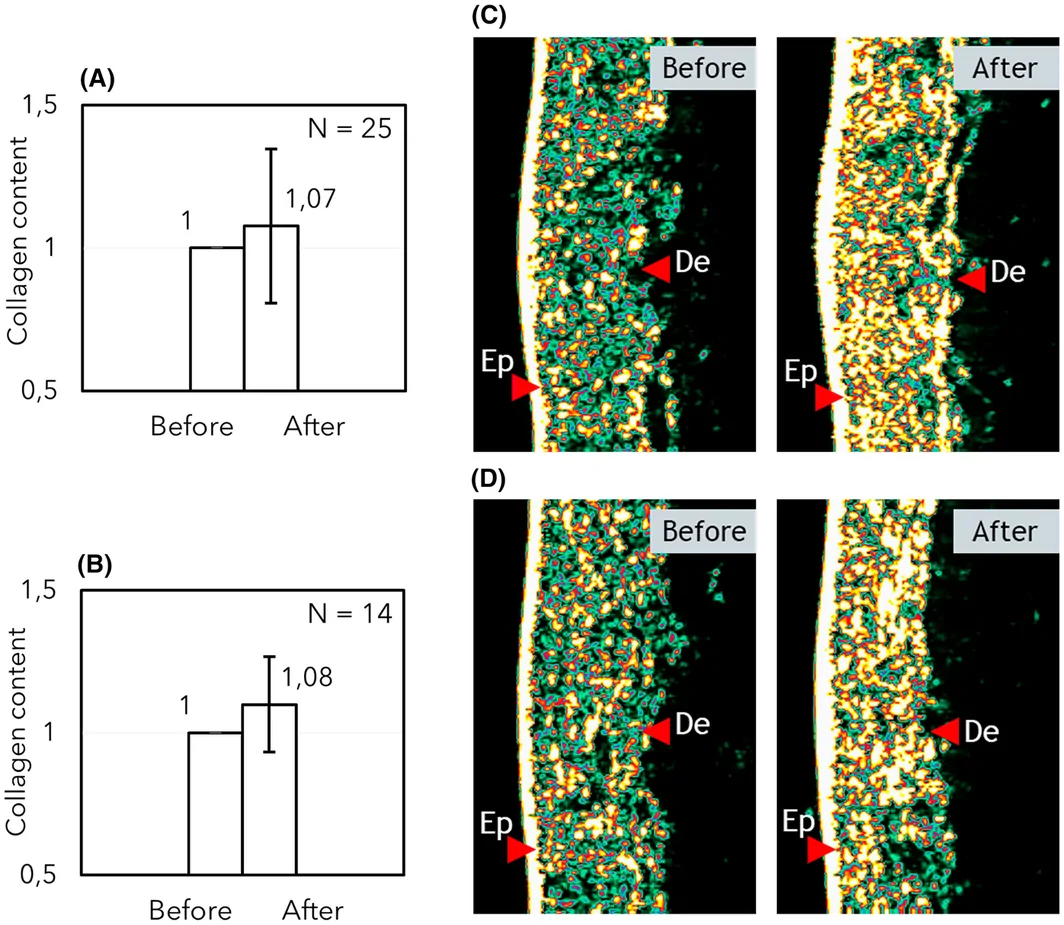
Ultrasound measurements of skin echogenicity (indirect indicator of collagen content). (A) Normalized values of DE in subjects treated in the abdomen/flanks. (B) Normalized measurements of skin echogenicity in thighs treatments. (C) Before and after ultrasound images of a subject treated in abdomen/flanks showing a 78% increase in DE (yellow). (D) Increase of 61% in DE (yellow) of a participant treated in the thighs. Ep: epidermis; De: dermis.

### 3.5 Visual effect in body contouring following Zionic™ treatment

Photographs from four different angles were taken in standardized position and lighting conditions. Following Zionic™ treatment, visual improvement was observed in most of the subjects. In general, the fat reduction and body contouring effect were more evident in the abdomen/flanks while the dermocorrection and reduction of skin irregularities were more noticeable in the thigh area (Figure 5).

**FIGURE 5:**
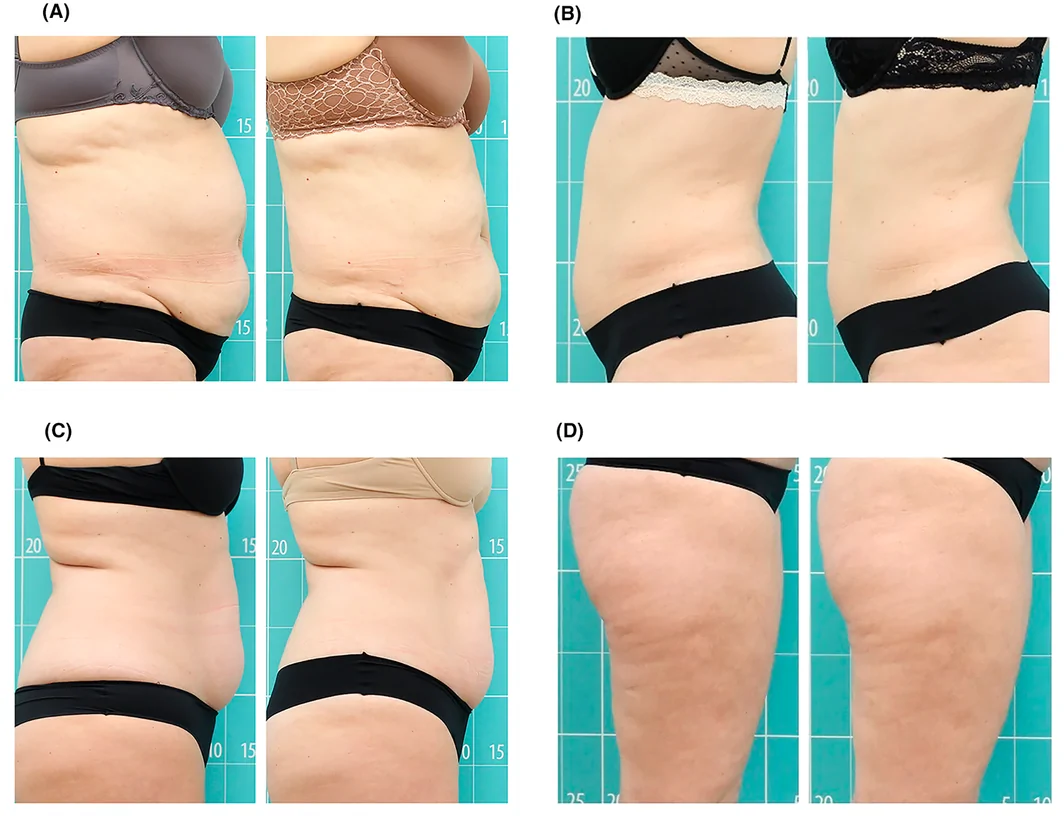
Clinical results of 4 representative participants following eight sessions of Zionic™. (A, B, and C) Volume reduction in abdomen/flanks and (D) visual improvement of skin appearance in thighs treatment.

### 3.6 Subjective surveys questions and adverse events report

Tolerance to the treatment was assessed after finishing each study. Zionic™ treatment was generally well tolerated. Minimal discomfort during treatment sessions resulting from skin heating were reported by 28.2% (*n* = 11) of the subjects. Some minor adverse effects occurred after treatment sessions: abdominal discomfort (*n* = 1), skin reaction (*n* = 1), stiff sensation (*n* = 1), nausea (*n* = 1), mild erythema (*n* = 1), and itching sensation in the treated area (*n* = 1). All of them were resolved within 24 h. A bruise associated to the treatment, a mild side effect, was detected in one participant. The reaction persisted fewer than 96 h and no sign was noted at the follow‐up session.

All subjects completed a satisfaction survey 1 month after the end of treatment. The questionnaires revealed that 82.05% (*n* = 32) of the participants were very satisfied (satisfaction level 7–10) with the outcomes of the treatment, 15.38% (*n* = 6) were satisfied with their results (satisfaction level 5–6), and 2.56% (*n* = 1) were not satisfied (satisfaction level 0–4). Furthermore, when participants were asked whether they would recommend the same treatment to others, 94.87% (*n* = 37) would do it.

## 4 DISCUSSION

Non‐invasive therapies for fat reduction and improvement of skin appearance are an aesthetic field that is constantly evolving. Currently, several devices are available deferring mainly on the source of energy or type of stimuli used to promote an activation of the target tissue. Radial shockwave‐therapy, cryolipolysis, and low level laser are among the most common technologies in the field.^14^, ^15^ However, given its high safety profile and efficacy in short treatments, noninvasive RF technology has emerged as the most promising technology of choice for both professionals and patients.

In recent years, manufacturers of noninvasive RF equipment for fat reduction, body contouring, and skin laxity improvement have tried to offer innovations such as the combination of technologies to enhance clinical performance. In this sense, the Zionic™ device (Termosalud Inc. Gijón, Spain) emerges as new generation RF equipment that combines electrothermal energy with mechanical stimulation of the tissue through a unique rotational monopolar electrode.

In this trial, we report a significant body contour and subcutaneous adipose tissue thickness reduction in the studied areas (abdomen/flanks and thighs) of 39 healthy volunteers. Following eight 45–50 min Zionic™ treatments, 72/96 h apart, participants showed a 3% reduction in abdomen/flanks contour (upper: 2.55 cm; middle: 2.50 cm and lower: 2.75 cm) and up to 4% reduction in the thigh contour (upper: 1 cm and lower: 2.12 cm). Subcutaneous adipose tissue thickness in both the abdomen/flanks and thighs was assessed using ultrasound probes and participants showed a significant 6% and 8% reduction, respectively. Consistently, these findings are supported by the data of other trials assessing the efficacy and safe use of monopolar RF technology in body contouring and fat tissue reduction. Anolik et al.^8^ using the monopolar ThermaCool TC System (Solta Medical Inc., Hayward, CA) obtained an average reduction in waist circumference of 1.4 cm. Similarly, in another randomized study, carried out by Somenek et al.^9^ the analysis of effectiveness and safety of the monopolar device TruSculptID (Cutera, Brisbane, CA) in adipose tissue thickness reduction was assessed. Following 1 session treatment, ultrasound analysis showed a significant 2.57 mm flank fat reduction. Considering studies involving devices with different handpiece architectures but based in similar adipose tissue heating objectives, the results were found to be similar to the ones observed in our study (3% for abdomen/flanks and 4% for thighs). Manuskiatti and colleagues (2009)^11^ used a tripollar RF device Regen™ (Pollogen) in a 8‐sessions treatment for abdomen and thighs, noting an average significant circumference reduction of 3.75% and 3.13%, respectively.

Zionic™ treatment was also found to be effective improving dermal structure. Echogenicity changes (assessed by high‐frequency ultrasound probes) are widely used to determine the aesthetic improvement of the skin. It offers high sensitivity and allows precise determination of the content and organization of the skin's collagen bundles (highly echogenic structures).^12^ In our study, DE increased in both treated areas (7% in abdomen/flanks and 8% in thighs; non‐significant), suggesting an improvement in the skin's extracellular matrix (ECM) structure. Additionally, a significant increase (6%) in dermal thickness was observed in the thighs, further supporting the skin‐enhancing effect of Zionic™ treatment. These results are consistent with the observations of Louis and colleagues (2020)^16^ who reported that RF‐induced dermal heating triggers the overexpression of Heat Shock Protein 47 (HSP‐47), an endoplasmic reticulum chaperone protein crucial for the proper assembly of new collagen molecules. HSP‐47 prevents collagen aggregation, an age‐related process that leads to collagen clumping and ECM disruption. By preventing aggregation, HSP‐47 ensures that collagen molecules are correctly incorporated into the ECM, contributing to skin firmness and resiliency.^17^, ^18^ Further molecular and histological studies have also established a link between the thermal enhancement of HSP‐47 synthesis and the induction of ECM proteoglycans and collagen Type XVII. Collagen XVII is a transmembrane protein that has been shown to play a crucial role in dermal‐epidermal adhesion, regulating epidermal cell proliferation, and consequently, the thickness of both youthful and aged skin.^16^, ^17^, ^18^, ^19^

Our results are also in line with others author's works that unveil the biological mechanisms triggered in the skin by RF. Boisnic et al.^1^ and Pinto et al.^20^ investigated the effects of RF in the hypodermis, where the adipocyte lipolytic metabolism is boosted and fat is significantly released to the bloodstream. Additionally, Roti et al. studied the molecular acute response to thermal stress that can trigger adipocyte apoptosis.^21^ Furthermore, RF‐mediated temperature increase enhances blood circulation and lymphatic drainage trough vasodilation, promoting the elimination of metabolic waste and the oxygenation of local tissues.^22^, ^23^ At the dermal level, electro‐thermal stimulation promotes ECM synthesis and its reorganization (including fibrous septa remodeling) which improve the appearance of the skin.^6^

Unlike other devices available in the market, the previously described biological effects are enhanced by the action of the innovative and patented rotational electrode. On the one hand, the mechanical movement provides an intense massage that contributes to the circulatory movement of released fat and the elimination of fluid build‐up.^24^ On the other hand, monopolar RF raises the temperature of the tissue to 41–42°C, activating neocollagenogenesis in the dermis and lipolysis in the hypodermis, allowing global remodeling work.^25^ This multi‐layer approach makes the Zionic™ treatment an excellent tool for body rejuvenation and contouring treatments.

In our study, we provide clinical evidence for the safety and efficacy of the novel RF device, Zionic™, in body contouring treatments. The observed results proved that dermal and fat tissue were stimulated leading to an improvement in skin properties and a reduction of fat tissue and body contour. Further clinical trials of this RF, with other skin types and histological assessments and/or male subjects will be considered, however, the current findings demonstrate a notable improvement following Zionic™ treatment protocol.

## 5 CONCLUSION

The findings show that the treatment is a safe, effective, and well‐tolerated noninvasive procedure for body contouring and improvement of skin appearance in thighs, flanks, and the abdominal area.

## AUTHOR CONTRIBUTIONS

All authors contributed to develop and review the article and agree with the final content. A.I.F. and L.S.F: recruitment, scheduling, performing treatments and safety supervisors. A.F.S, L.H.Z and S.V.F: recruitment, study design, research monitoring, data collection, data analysis, interpretation, and paper writing.

## CONFLICT OF INTEREST STATEMENT

The research team for this study is composed of Termosalud, Inc. (Gijón, Spain) personnel. The research was overseen by the Asturias Ethics Committee and an authorized Clinical Research Associate (CRA) to ensure that the experiments and interpretation of results were not influenced by any commercial interests. All the clinical trial treatments were performed in an aesthetic center associated to Termosalud company. The authors of the paper are employed by Termosalud S.L and are responsible for the content of the article. The results were not influenced by personal interests of the research team or the company.

## ETHICS STATEMENT

The study, approved on July 19th, 2022, with the code 2022.252, was conducted in accordance with the criteria established by the Research Ethics Committee of the Principality of Asturias (1964 Declaration of Helsinki and its later amendments). Written informed consent and photographic consent were obtained from all the subjects for their inclusion in the study and publication of their anonymized data.

## Supporting information


Data S:

## Data Availability

The data that support the findings of this study are available from the corresponding author upon reasonable request.
