# Correction to “Rotational Radiofrequency‐Based Technology Leads to Adipose Tissue Reduction and Contouring Effect in the Thighs, Abdomen, and Flanks”

**DOI:** 10.1111/jocd.70190

**Published:** 2025-04-21

Santos AF, Fernández AI, Fernández LS, Zapico LH, Freitag SV. Rotational Radiofrequency‐Based Technology Leads to Adipose Tissue Reduction and Contouring Effect in the Thighs, Abdomen, and Flanks. *Journal of Cosmetic Dermatology*. 2024; 23: 3263–3271. http://doi.org/10.1111/jocd.16431.

In the “Results” section of the “Abstract” of the reference article, the text “Abdomen/flanks and thighs subcutaneous fat layer thickness was significantly reduced by 8% and 6%, respectively” was incorrect. This has been corrected as follows: “Abdomen/flanks and thighs subcutaneous fat layer thickness was significantly reduced by 6% and 8%, respectively.”

We apologize for this error.

