# Supplementary material for: Rotational radiofrequency‐based technology leads to adipose tissue reduction and contouring effect in the thighs, abdomen, and flanks
Source: J Cosmet Dermatol. 2024 Jul 1;23(10):3263–71. doi: 10.1111/jocd.16431 (PMC13649086; doi:10.1111/jocd.16431)
Supplement: Supplementary file 1 [file JOCD-23--s001.docx]

**SUPPLEMENTARY MATERIAL**

**1-. List of Zionic contraindications**

- Pregnancy and breastfeeding. Pregnancy is a contraindication in the application of radiofrequency treatments. The person should not be pregnant at the time of starting the treatment and should avoid becoming pregnant during the whole period in which the sessions are carried out.

- Coagulation disorders, or anticoagulant medication.

- Uncontrolled hypertension

- Diabetes mellitus

- Phlebitis or vasculitis

- Cancer or tumours

- Obesity

- Vascular fragility

- Recent surgery (6 weeks)

- Infections, irritated or eroded areas or open wounds on the skin in the area to be treated

- Varicose veins (in the treatment area)

- Medical conditions such as: heart, lung, or circulatory disease

- Inability to understand the consequences, implications and risks of the procedure

- High body temperature (pyrexia)

- Presence of metallic implants or prosthesis in the area

- Presence of metallic IUD (if treatment is performed in the abdomen)

- Pacemaker wearers

- Epilepsy

- Absence of skin sensitivity

- Medication

- Concomitant devices.

**2-. Patient flow diagram (CONSORT)**

Figure 1. Flowchart of the subjects involved in the clinical trial (CONSORT).

**3-. Clinical measurements**

Table 1. Summary of measurements taken in the treatment areas (abdomen/flanks or thighs) of the 39 subjects involved in the trial.

|  | Abdomen/flanks  (n) | Thighs  (n) | Total  (n) |
| --- | --- | --- | --- |
| Body contours | 75 | 28 | 103 |
| Upper | 25 | 14 | - |
| Middle | 25 | * | - |
| Lower | 25 | 14 | - |
| Skin echogenicity | 75 | 42 | 117 |
| Skin thickness | 75 | 42 | 117 |
| Fat layer thickness | 75 | 42 | 117 |
| Photographs | 100 | 56 | 156 |

* Contours at the middle height were not measured in thighs procedures.

**4-. Circumference and ultrasound evaluation protocol**

Figure 1. Anatomical localization of body contours measuring heights () and ultrasound measuring points ( ). (A) Abdomen/Flanks circumferences and ultrasound scans were taken at 3 different heights and at 3 points. (B) Thighs circumferences were measured at 2 different heights and the ultrasound scans at 3 different points. Coordinates of the studied heights/points were noted: height from the floor and distance from an anatomical reference axis (the dawn line for treatments in abdomen/flanks and the popliteal line for treatments in thighs).

**5-. Satisfaction survey**

1. Indicate your gender.

| Man | Woman | Other (specify) |
| --- | --- | --- |

2. Indicate your age.

| <25  26-35  36-45 | 46-55  56-65  > 65 |
| --- | --- |

3. What was the reason you underwent this treatment? You may indicate more than one option.

| Localized fat reduction | Body contour remodeling | Anti-cellulite |
| --- | --- | --- |

4. In what area was the treatment performed? You may indicate more than one option.

| Abdomen  Flanks  Thighs | Buttocks  Other. Specify where. |
| --- | --- |

5. From 1 to 10, how satisfied are you with the results obtained after the treatment with Zionic^TM^ device?

1	2	3	4	5	6	7	8	9	10

6. From 1 to 10, how do you valorate the attention received from the treatment performers?

1	2	3	4	5	6	7	8	9	10

7. From 1 to 10, how satisfied do you feel with the information provided about the treatment performance prior to its beginning?

1	2	3	4	5	6	7	8	9	10

8. Did you experience any of the following adverse event? Mark the corresponding ones.

| Pain  Erythema (skin redness)  Burns | Allergy  Bruising  Blood pressure drop  Other. Specify which one. |
| --- | --- |

9. If you have suffered discomfort during the performance of the treatment, how would you rate it from 1 to 10?

1	2	3	4	5	6	7	8	9	10

10. Would you recommend the treatment to other people?

Yes	No

11. When did you begin to notice the positive effects of the treatment?

After the first session	After the fifth session

After the second session	After the sixth session

After the third session	After the seventh session

After the fourth session	After the eighth session

Other (describe)

12. Had you previously heard of the capacitive and resistive radiofrequency technique?
13. What were your expectations of the treatment prior to its beggining?
